# PPP2R2B downregulation is associated with immune evasion and predicts poor clinical outcomes in triple-negative breast cancer

**DOI:** 10.1186/s12935-020-01707-9

**Published:** 2021-01-06

**Authors:** Zheng Li, Yaming Li, Xiaolong Wang, Qifeng Yang

**Affiliations:** 1 Department of Breast Surgery, General Surgery, Qilu Hospital of Shandong University, Jinan, China; 2 Pathology Tissue Bank, Qilu Hospital of Shandong University, Jinan, China; 3 Research Institute of Breast Cancer, Shandong University, Jinan, Shandong China

**Keywords:** PPP2R2B, Immune evasion, Bioinformatics, Prognosis, TNBC

## Abstract

**Background:**

Although immune checkpoint blockade has emerged as a novel promising strategy for triple-negative breast cancer (TNBC), many patients fail response or acquire resistance to current agents. Consequently, our focus need to shift toward alternative inhibitory targets, predictor for responsiveness, and immune suppressive mechanisms.

**Methods:**

In this study, we performed systematic bioinformatics analyses to identify PPP2R2B as a robust tumor suppressor in TNBC. Meanwhile, breast cancer progression cell line model was applied in our research. Quantitative real-time PCR assay (Q-PCR) was carried out to assess the role of PPP2R2B in the onset and progression of breast cancer. Furthermore, we validated the effect of PPP2R2B on immune activity via in vitro experiments based on macrophages. To further decipher the roles of PPP2R2B in TNBC, we investigated the transcriptome level, genomic profiles, and its clinical prognostic value.

**Results:**

In TNBC tissues, PPP2R2B expression was significantly downregulated compared to normal breast tissues. Kaplan‐Meier survival analysis revealed that patients with low PPP2R2B expression had shorter survival time than those with high PPP2R2B expression. Q-PCR analysis suggested that PPP2R2B downregulation could play a key role in breast-cancer initiation and progression. Additionally, our findings showed that PPP2R2B was positively related with CD8 T cells, CD4 Th1 helper cells, and M1 macrophages, but negatively related with M2 macrophages. Subsequent results identified that PPP2R2B was strongly related with immune inhibitor genes (GZMA, PRF1, and IFNG), which could improve T lymphocytes antitumor function and restrict immune evasion. Meanwhile, T cell receptor signaling pathway and antigen processing and presentation signaling pathway were significantly suppressed in low PPP2R2B expression group. Afterwards, distinct subgroups based on PPP2R2B expression exhibited several unique features in somatic mutations, copy numbers alterations, extent of copy number burden, and promoter methylation level.

**Conclusion:**

Our results indicated that PPP2R2B could serve as a promising biomarker for TNBC, and help predict immunotherapeutic response and guide personalized strategies in TNBC treatment.

## Background

Breast cancer is regarded as a common malignant disease in women worldwide [1]. In breast cancer molecular subtypes, triple-negative breast cancer (TNBC) carrying the worst prognosis is defined by the lack of estrogen receptor (ER), progesterone receptor (PR), and human epidermal growth factor receptor 2 (HER2) [2]. Despite the adjuvant treatment has improved in recent years, the treatment of TNBC is still up against several challenges owing to the absent expression of targetable receptors and heterogeneous clinical behavior [3]. Currently, immunotherapy have represented a promising therapeutic strategy for several cancer types, including breast cancer [4–6]. Compared to other breast cancer subtypes, TNBC could harbor greater potential in immunotherapy due to higher levels of tumor infiltrating lymphocytes (TILs), programmed death-ligand protein (PD-L1), and nonsynonymous mutations [7, 8]. However, the recurrence and metastasis of TNBC are frequently facilitated by immune evasion, which is partly due to the restriction of anti-tumor immunity derived by T-lymphocyte in the tumor microenvironment [9].

Cytotoxic T lymphocytes (CTLs) are considered as major effectors of immunity, and play a pivotal role in cancer immunotherapy [10]. Distinct subsets of tumor infiltrating T cells hold diverse functional properties in immune response. In tumor infiltrating T lymphocytes cells, CD8 T and CD4 Th1 cells typically contribute to immune-mediated tumor suppression [11]. In particular, CD8 T cells are regarded as a central player in restraining tumor progression [12, 13], which exert the role of tumor suppression mainly by perforin-granzyme, Fas-FasL, ferroptosis, and pyroptosis [14]. Additionally, CD4 Th1 subsets could orchestrate anti-cancer immunity and enhance the activation and development of tumor-specific CD8 T cells by producing IFN-γ, TNF-α, and interleukin-2 [15]. Thus, it is a desirable approach for improving patients’ clinical outcomes to escalate the effector of T lymphocytes-mediated anti-tumor immunity.

Phosphatases involved in several biology process, including immune response [16]. Previous reports showed that PP2A could activate T cells response by repressing CTLA-4 function or impairing expression of PD-L1 [17]. Protein phosphatase 2A (PP2A) belongs to the Ser/Thr protein phosphatase family, which is comprised by a scaffold A subunit, a highly conserved catalytic C subunit, and different regulatory B subunits [18].Of note, distinct regulatory subunits but not the catalytic subunit lead to special biological outcomes of PP2A [19]. PPP2R2B gene could encode regulatory subunit B55β to form PP2A-B55β complex via binding to the scaffolding and catalytic subunits. Jing Tan et al. reported that PPP2R2B inactivation could target PDK1/MYC signaling to promote colorectal cancer cells growth and contribute to rapamycin resistance [19]. Iris K Madera-Salcedo et al. [20] confirmed that PPP2R2B could prevent organ damage from activated T cells in chronic inflammation derived from systematic autoimmune diseases. Hyper-methylation of PPP2R2B induced acquired apoptosis deficiency and contribute to autoimmune diseases. Additionally, PPP2R2A gene was proved to produce regulatory subunit B55 to synthesize PP2A-B55 complex, and its deletion contributed to breast cancer cell proliferation via upregulating MASTL expression [21]. Moreover, PP2A was also found to promote ER re-expression in the ER-negative cell lines, indicating its potential clinical value for reversing tamoxifen resistance in TNBC patients [17]. However, the immune role of PP2A in TNBC still remain largely unknown.

In this study, we identified PPP2R2B as a robust tumor suppressor, and played an important role in anti-tumor immune response, which dysregulation could contribute to the onset and progression of breast cancer. Afterwards, we evaluated the biological roles of PPP2R2B using systematic bioinformatics approaches and in vitro experiments. Finally, our results demonstrated that PPP2R2B could provide potential clinical benefits for TNBC patients treated with immunotherapy in the future.

## Materials and methods

### Sample datasets

TCGA level3 RNA-seq dataset was obtained and integrated by TCGAbiolinks package [22] from The Cancer Genome Atlas data portal (https://portal.gdc.cancer.gov/). METABRIC dataset was sourced from Molecular Taxonomy of Breast Cancer International Consortium (https://www.mbcproject.org/). GSE21653 and GSE1456 datasets were obtained from Gene Expression Omnibus (https://www.ncbi.nlm.nih.gov/gds/). The inclusion criteria included survival time, survival status, age, TNBC patients (basal-like subtype or ER/PR/ HER2-negative), clinical stage (TCGA), follow-up time not less than 1 month. As a result, 147 cases of TCGA, 297 cases of METABRIC, 74 cases of GSE21653, and 25 cases of GSE1456 were enrolled into this study. The ids of included patients were listed in Additional file 1: Table S1.

147 cases with copy number alterations and 141 cases with somatic mutations were downloaded via TCGAbiolinks package, all of which were corresponded to the TNBC cases with RNA-seq data. Copy number alterations were analyzed by GISTIC2.0 [23]. Somatic mutations analysis was performed by Maftools package [24]. DNA methylation data was derived from 450 K Methylation array data. CpG island probes located in gene promoter region was extracted via the ChAMP package [25]. Promoter methylation level was evaluated by MEXPRESS (https://mexpress.be/).

### Cell lines and cell culture

All cell lines used were obtained from the American Type Culture Collection (Manassas, VA) and cultured in a 5% CO2-humidified incubator at 37 °C following the manufacturer’s instructions. MDA-MB-231 and MDA-MB-468 cells were cultured in Dulbecco’s modified Eagle’s medium (Invitrogen, USA). THP-1 cells were cultured in RPMI 1640 medium. The medium used above was supplemented with 10% fetal bovine serum (Hyclone), 100 U/ml penicillin, and 100 μg/ml streptomycin.

### Macrophage polarization

Briefly, THP-1 monocytes were differentiated into M0 macrophages by treatment with 150 nM PMA for 24 h. After 24 h, M0 macrophages were polarized into M1 macrophages by stimulation with LPS (100 ng/mL) and IFN-γ (20 ng/mL) for 48 h. Conversely, M2 phenotype polarization was obtained by stimulation with IL-4 (20 ng/mL) and IL-13(20 ng/mL) for 72 h.

### Weighted gene correlation network analysis (WGCNA)

First, TCGA level3 RNA-seq dataset was normalized by DESeq2 [26] and preprocessCore packages. Additionally, genes with zero variance between high- and low- groups was removed, and the first 50% genes with median absolute deviation (MAD) value at least greater than 0.01 were retained. In this study, WGCNA package [27] was used to define 8 as optimum soft threshold power and construct a scale-free network and topological overlap matrix (TOM). DeepSplit of 2 and minModuleSize of 30 was set as parameters of the Dynamic Tree Cut method to avoid generating too many modules. Height cut-off value was identified as 0.25 to merge modules with similarity > 0.75. Finally, the enrolled genes generated 13 modules (except the grey module) by cluster analysis. According to Pearson correlation analysis, we evaluated the association between module eigengenes(MEs) and infiltrated T lymphocytes, and then identified blue module as the highly related module. The key genes were defined by module membership(MM) greater than 0.8 and gene significance(GS) greater than 0.5.

### Primary identification of the hub prognostic genes associated with lymphocytes infiltration

Based on the key genes screened by WGCNA, an immune signature score was developed using single sample Gene Set Enrichment Analysis (ssGSEA). Next, Spearman correlation analysis was separately performed to identify the genes highly correlated with the immune signature score in TCGA and METABRIC datasets, of which 883 were overlapped (Additional file 2: Table S2). After primary filter, the 611 survival-related genes (Additional file 3: Table S3) were sent to Lasso penalized Cox regression analysis. Across 1000 iterations analysis with cross-validation, we finally screened out 4 hub prognostic genes. The analysis procedure was conducted by the previous study [28].

### Gene ontology analysis (GO), Gene set variation analysis (GSVA), and Gene set enrichment analysis (GSEA)

The clusterProfiler package [29] was performed to investigate the biological process of the related genes. GSEA was performed by the JAVA program using the Kyoto Encyclopedia of Genes and Genomes (KEGG) gene sets obtained from MSigDB. And all genes were ranked on the basis of differential significance between the high- and low- level subgroups stratified by the quartile of PPP2R2B expression. After performing 1000 permutations, gene sets enrichment with nominal *P* < 0.05 and FDR < 0.25 were considered significant. In Gene set variation analysis, GO gene sets were sourced from AmiGO 2 Web portal (http://amigo.geneontology.org/amigo/landing). Spearman correlation analysis was carried out to assess the relationship between PPP2R2B expression and specific immune gene sets.

### RNA extraction, reverse transcription, and quantitative real-time PCR (Q-PCR) assays

Total RNA from breast cancer cells was extracted using The TRIzol reagent (Invitrogen). Complementary DNA (cDNA) of mRNA was reversely transcribed using PrimeScript reverse transcriptase (RT) reagent kit (TaKaRa, Shiga, Japan). Biosystems StepOne plus System was employed to perform Q-PCR assay. Primers used for Q-PCR in this study are listed in Additional file 4: Table S4.

### Immunohistochemistry(IHC)

Paraffin-embedded specimens were sliced into 4-μm sections, followed by a 60 °C bake for 1 h. Then sections were deparaffinized with xylene, rehydrated via a graded alcohol series, and retrieved in EDTA antigenic retrieval buffer using microwave heating. After a 20 min cooling at room temperature, the endogenous peroxidase activity was quenched with 3% hydrogen peroxide, and then nonspecific antigen binding was blocked with normal serum at 37 °C for 1 h. The sections were incubated with rabbit anti-PPP2R2B (Proteintech, 1:100) at 4 °C overnight. After treatment with biotinylated anti-rabbit secondary antibody at 37 °C for 1 h, the sections were treated with horseradish peroxidase (HRP)-conjugated streptavidin, stained with diaminobenzidine (DAB), and counterstained with hematoxylin. Images were obtained using an Olympus light microscope. In this study, formalin-fixed and paraffin-embedded human breast biopsy specimens were sourced from the archives of the Department of Pathology at Qilu hospital of Shandong University.

### Immunofluorescence (IF)

For immunofluorescence, cells cultivated on glass cover slides were rinsed in PBS three times, fixed with 4% paraformaldehyde for 10 min, permeabilized in 0.3%Triton X-100 for 20 min, and blocked in goat serum solution (PBS containing 10% goat serum and 0.3% Triton X-100) for 1 h at room temperature. Following blocking, cells were incubated with rabbit anti-PPP2R2B (Proteintech, 1:100) overnight at 4 °C. Then secondary antibody (Invitrogen, Goat Anti-Rabbit IgG Alexa Fluor 488,1:100) incubated for 2 h at room temperature. Lastly, the nucleic acid was counterstained with DAPI, and fluorescent microscopy was used to observe the treated cells.

### Western blotting assay

Proteins were isolated from collected cells using western lysis buffer containing protease inhibitors. After protein concentration was quantified using the BCA method, 50 μg of proteins from each sample were loaded and separated on 10% SDS-PAGE gels. Subsequently, proteins were electrotransferred onto a PVDF membrane (Bio-Rad, Hercules, CA, USA) at 200 mA for 2 h. Then 5% non-fat milk was used to block non-specific binding sites for 1 h. After overnight incubation with specific antibodies at 4 °C, the members were incubated with the appropriate secondary antibodies for 2 h at room temperature. The protein bands were visualized by enhanced chemiluminescence (ECL; Bio-Rad, USA). β-Actin protein expression was used as a loading control. The primary antibodies used in this study were purchased from Proteintech Group.

### Transwell assay

In vitro migration ability was assessed using a migration assay, which was performed using transwell inserts (8-μm pore size, Corning Costar, USA) in 24-well plates (Corning Costar, USA). 1 × 10^5^ MDA-MB-231, 1.5 × 10^5^ MDA-MB-468 or 1.5 × 10^5^ macrophages were suspended in 200μL serum-free medium and seeded into the inside of each insert, while 700μL medium containing 20% FBS was placed in the lower well. After incubation for 24-48 h, the infiltrating cells, on the lower surface, were fixed with methanol and stained with 0.1% crystal violet. Following the infiltrating cells were photographed, ImageJ software was used to count the number of cells.

### Statistical analysis

CIBERSORT deconvolution method [30] with leukocyte signature matrix (LM22) and standard settings was conducted to estimate the fraction of tumor infiltrating immune cells in each tumor sample with TCGA RNA-seq data via voom transformation as input. In this analysis, breast cancer patients with P-values less than 0.05 were selected to further identify the immune landscape. Kaplan-Meier survival curve based on log-rank test was drawn by survminer package. Univariate Cox regression analysis was performed by Survival package. The time‐dependent receiver operating characteristic (ROC) analysis was conducted using the survivalROC package. Experiment data were presented as the mean ± SD (standard deviation). Continuous data between two groups were assessed using Student's t-test or Wilcoxon test, otherwise the one-way ANOVA or Kruskal–Wallis test. A probability value (*p*) < 0.05 was considered as significant difference. In this study, R project (Version 3.6.1) and GraphPad Prism 8 were used to perform the main statistical analysis.

## Results

### Construction and evaluation of the immune signature in TNBC

**Fig. 1:**
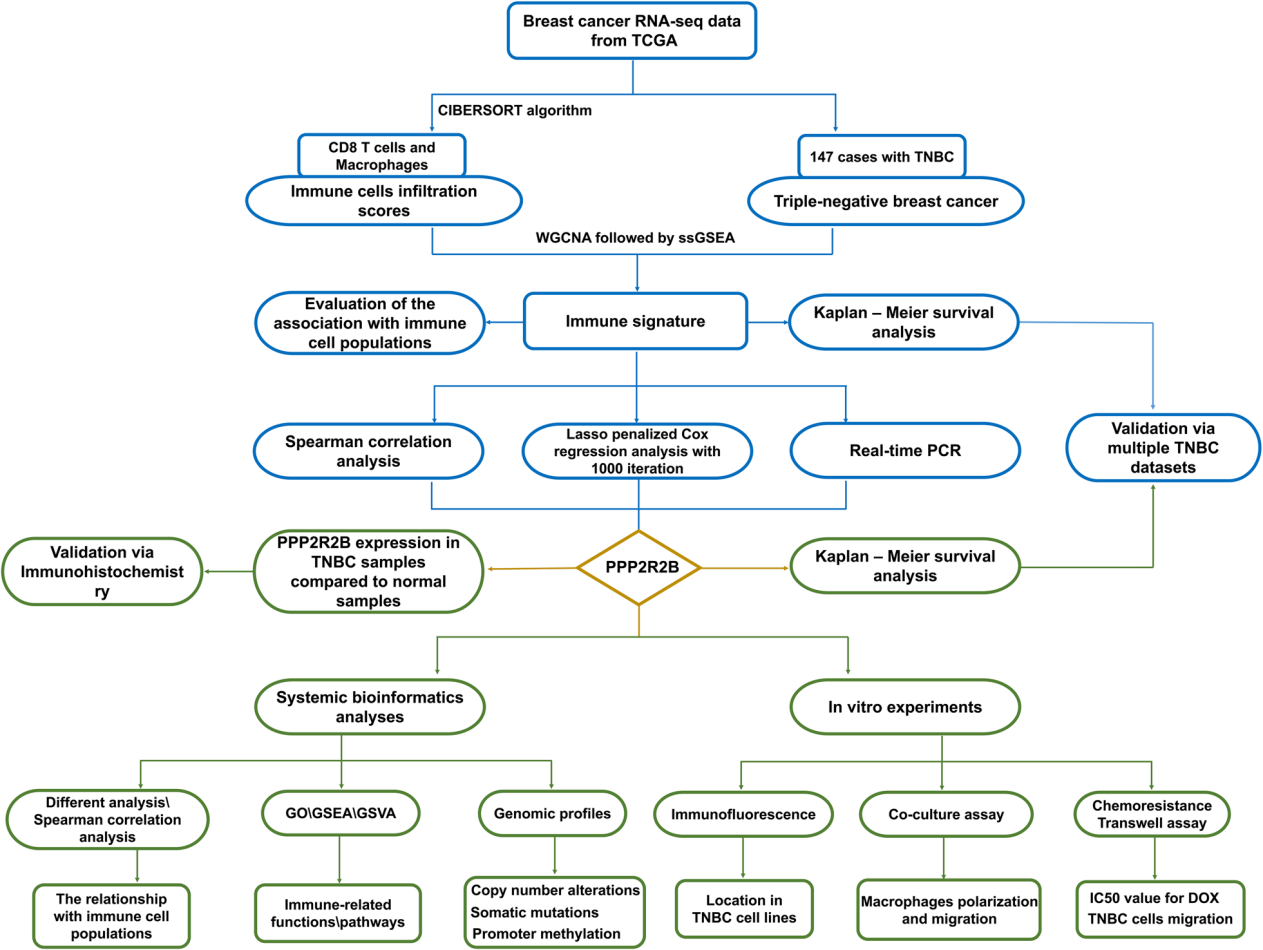
A flowchart depicting our study procedure

**Fig. 2:**
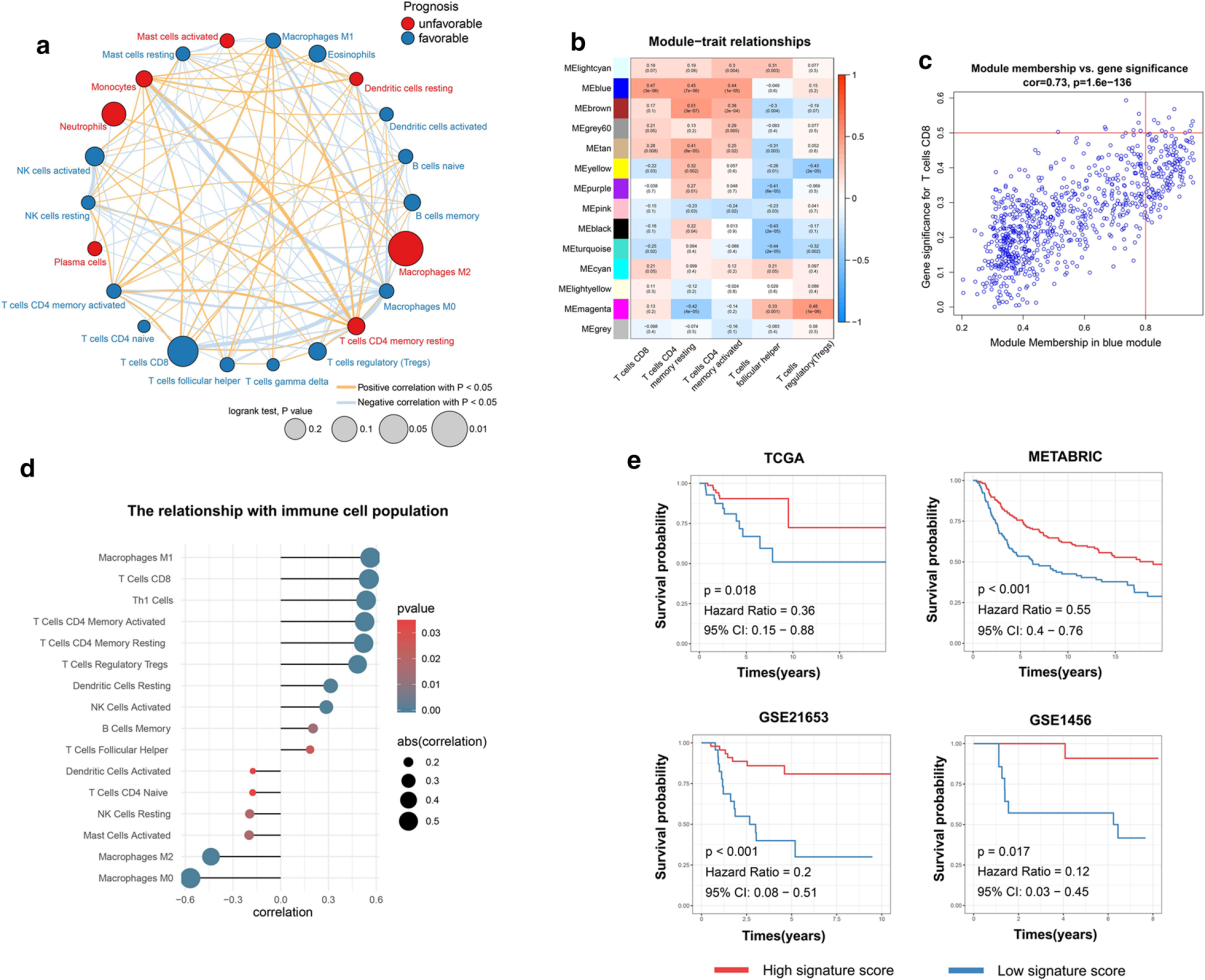
Construction and evaluation of the immune signature in TNBC. **a** The immune landscape of breast cancer. **b** Module‐trait associations were evaluated by correlations between MEs and clinical traits. **c** A scatter plot of GS for infiltration signature vs MM in blue module. red line represents the screening criteria: MM value greater than 0.8 and GS value greater than 0.5. **d** The relationship between immune cell populations and immune signature. **e** Kaplan‐Meier survival analysis in multiple TNBC datasets

As shown in Fig. 1, a flowchart was used to depict our study procedure. Figure 2a exhibited a comprehensive immune landscape of breast cancer, which mainly described immune cell interactions, cell lineages, and their effects on the overall survival of patients with breast cancer. CD8 T cells and M2 Macrophages showed significant difference in the univariate Cox regression analysis. Consistent with previous studies, CD8 T cells predicted a good prognosis, whereas M2 Macrophages predicted a poor prognosis. In this study, we focused on CD8 T cells due to its central role in immune surveillance and immunotherapy. To better identify TNBC-specific biomarker associated with CD8 T cells infiltration, we performed WGCNA to obtain the crucial module highly related to CD8 T cells infiltration (Fig. 2b–c; Additional file 6: Figure S2a). Finally, we confirmed six crucial prognostic genes as CD8 T cells-related genes in TNBC, including CD3D, CD3E, CD247, GZMA, CRTAM, and SLA2 (Additional file 5: Figure S1a–f). Subsequently, ssGSEA was carried out to construct an immune signature on the basis of the above genes, which performance was evaluated by Spearman correlation analysis (Fig. 2d). Interestingly, the immune signature not only had a significantly positive relationship with CD8 T cells but also strongly positive association with M1 macrophages, whereas it was significantly negatively related to M0 macrophages and M2 macrophages. Hence, we speculated that the immune signature may reflect macrophage polarization state within breast cancer microenvironment. Moreover, the patients with high scores had longer survival time than the patients with low scores in multiple TNBC datasets (Fig. 2e).

### PPP2R2B was identified as a hub prognostic biomarker and associated with malignant progression of breast cancer

**Fig. 3:**
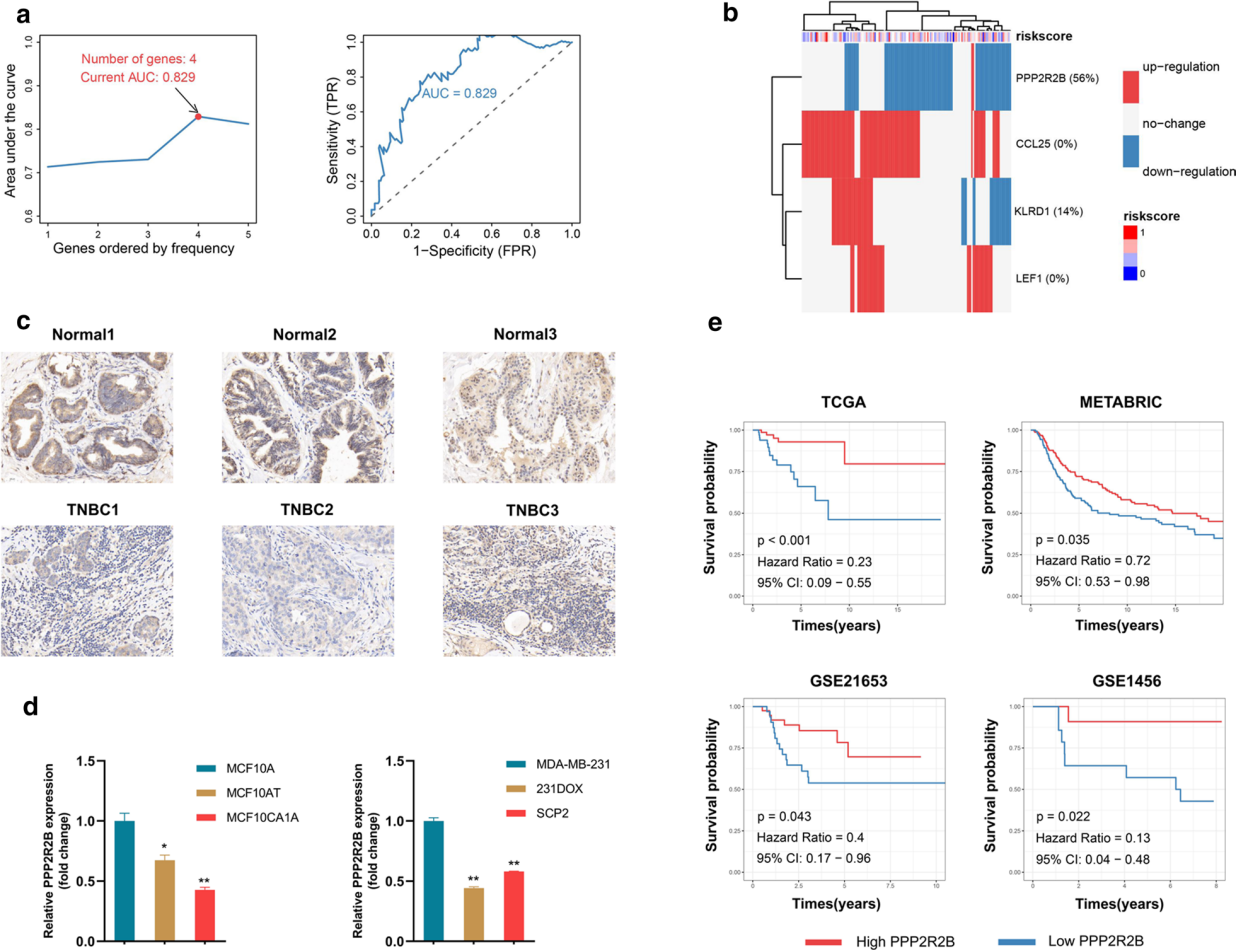
PPP2R2B was identified as a hub prognostic biomarker and associated with malignant progression of breast cancer. **a** Through Lasso penalized Cox regression analysis with 1000 iterations, a signature containing 4 genes was identified as an optimal signature with maximum 5 year AUC value. **b** The expression difference of 4 signature genes between breast normal tissues and TNBC tissues in TCGA. **c** Immunohistochemistry staining of PPP2R2B expression in breast normal tissues and TNBC tissues. **d** Quantitative real-time PCR analysis. **e** Kaplan‐Meier survival analysis of PPP2R2B in multiple TNBC datasets. *ns* Not significant, *p < 0.05, **p < 0.01, ***p < 0.001 and ****p < 0.0001

To further explore robust biomarkers for predicting survival and immune infiltration status, the highly related genes with immune signature score in TCGA and METABRIC datasets were sent to the following analysis (Additional file 6: Figure S2b, c). After the primary filter, Lasso penalized Cox regression analysis was conducted to assess optimal predictive signature on the filtered dataset. The 4 genes signature with optimal AUC value was confirmed after 1000 iterations analyses (Fig. 3a). Our finding revealed that PPP2R2B appeared more frequently than other genes across iterations analysis (Additional file 6: Figure S2d). In TCGA, PPP2R2B expression showed significant downregulation in TNBC samples compared to normal breast samples (Fig. 3b). We then used IHC to examine PPP2R2B expression in normal breast tissues and TNBC tissues (Fig. 3c). Indeed, the staining intensity of PPP2R2B was observed to be significantly decreased in TNBC tissues compared to normal breast tissues. Meanwhile, the staining distribution showed that most of the PPP2R2B was located in cytoplasm in normal breast tissues and TNBC tissues. To investigate the roles of PPP2R2B in breast cancer, breast cancer progression cell line model (MCF10A\MCF10AT\ MCF10CA1A) was applied in this research. Q-PCR assay demonstrated that PPP2R2B gene expression exhibited a trend of gradient downregulation from MCF10A to MCF10AT and MCF10CA1A cells (Fig. 3d). Moreover, compared with parental TNBC cell line (MDA-MB-231), PPP2R2B downregulation was detected in doxorubicin-resistant TNBC cell line (231DOX) and bone metastatic TNBC cell line (SCP2) (Fig. 3d). Based on the Q-PCR assay, we speculated that PPP2R2B may participate in the development and progression of breast cancer. Additionally, decreased PPP2R2B expression was associated with poor survival time, which was validated in multiple TNBC datasets (Fig. 3e).

### The relationship between PPP2R2B expression and infiltrated immune cell populations

**Fig. 4:**
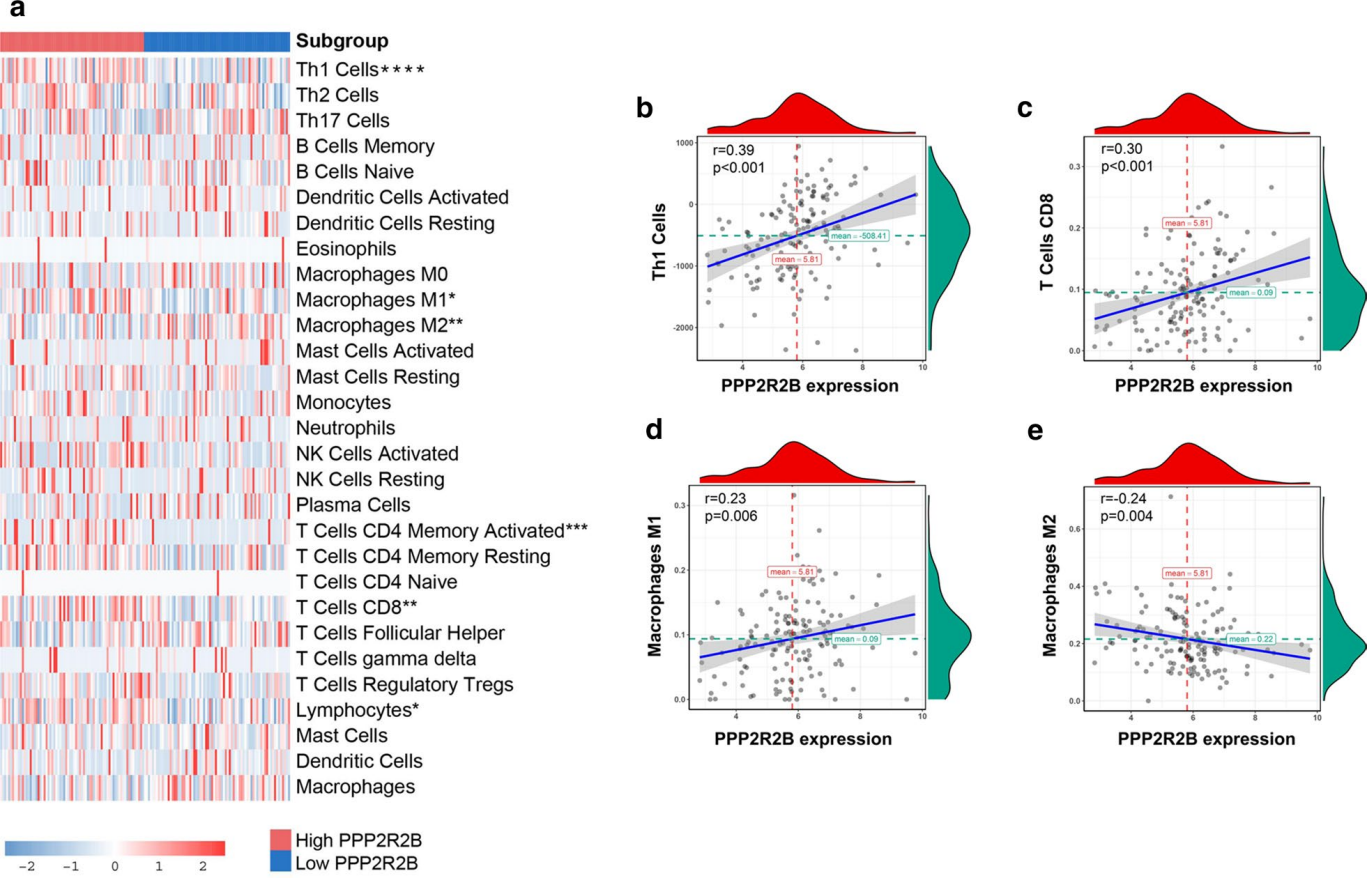
The relationship between PPP2R2B expression and infiltrated immune cell populations. **a** The infiltration levels of immune cell populations in TNBC patients with different PPP2R2B expression. **b**–**e** Spearman correlation analysis. CD4 Th1 cells, CD8 T cells, M1 macrophages, and M2 macrophages, respectively. *ns* Not significant, *p < 0.05, **p < 0.01, ***p < 0.001 and ****p < 0.0001

The previous immune infiltration dataset [31] was employed to assess the relationship between PPP2R2B expression and infiltrated immune cell populations. TNBC patients derived from TCGA were stratified into two subgroups based on PPP2R2B median expression value. Infiltrated immune cells with significant difference were confirmed by Wilcoxon test (Fig. 4a). The results showed that CD4 Th1 cells, M1 macrophages and CD8 T cells were significantly enriched in high expression group, while M2 macrophages infiltration was activated in low expression group. Spearman correlation analysis was conducted to verify the above relationship, which revealed that PPP2R2B expression was positively associated with CD4 Th1 cells, M1 macrophages, and CD8 T cells, and negatively associated with M2 macrophages infiltration (Fig. 4b–e).

### The immune functions of PPP2R2B in TNBC

**Fig. 5:**
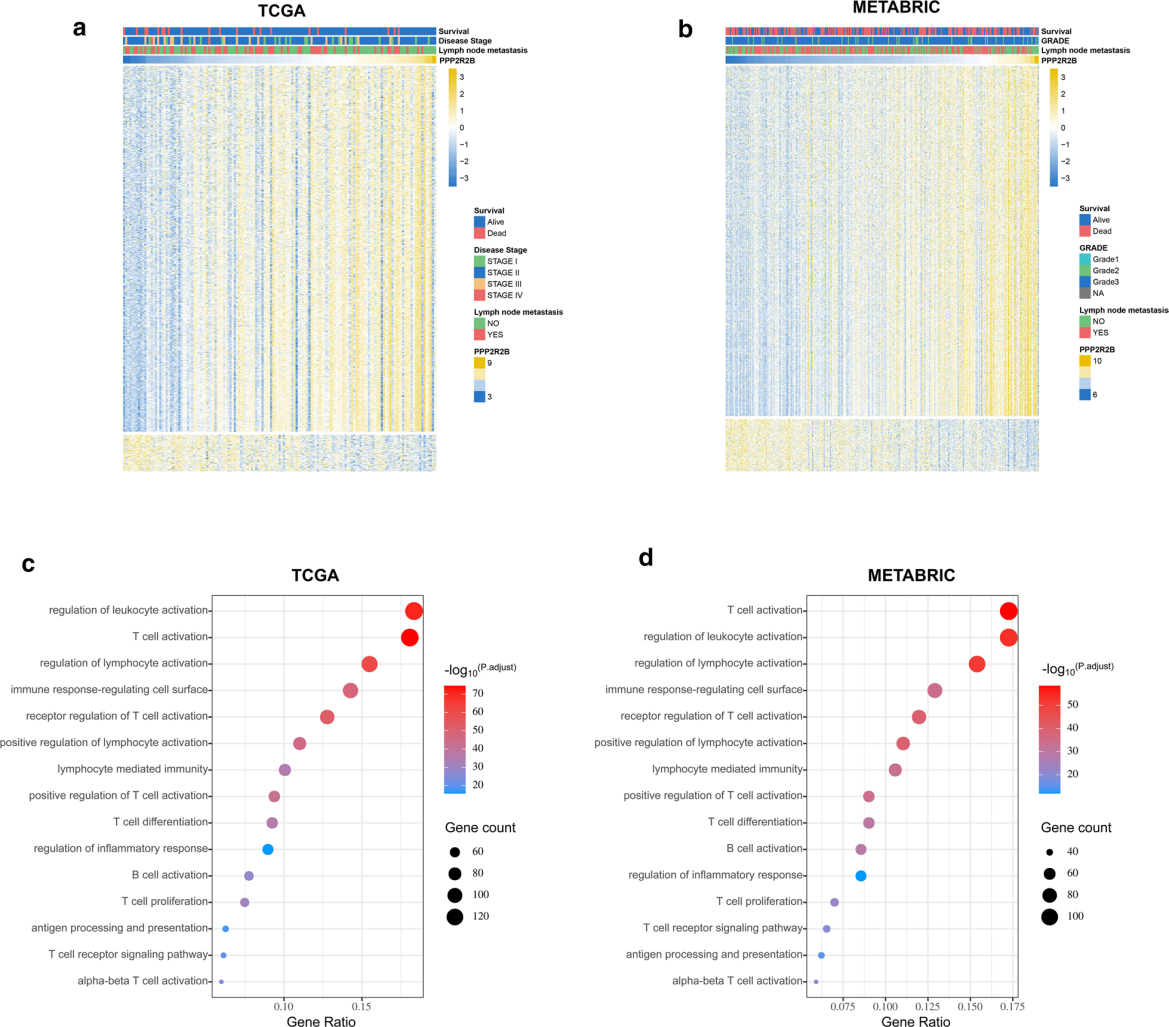
Gene ontology analysis for PPP2R2B in TNBC. **a**, **b** Heat maps showed that the most related genes with PPP2R2B expression in TCGA and METABRIC, respectively. **c**, **d** Biological processes of positively related genes with PPP2R2B expression in TCGA and METABRIC, respectively

The heat maps (Fig. 5a, b) exhibited the genes with Spearman |R|> 0.3 in TCGA and METABRIC datasets, respectively. To further elucidate the biologic functions of PPP2R2B in TNBC, genes correlated with PPP2R2B expression (Spearman R > 0.3) in TCGA (821genes; Additional file 7: Table S5) and METABRIC (779genes; Additional file 7: Table S5) were submitted to GO analysis. Our findings revealed that the most genes were associated with T cell activation and regulation, inflammation response and antigen processing and presentation in TCGA dataset (Fig. 5c). Importantly, the similar result was observed in METABRIC dataset (Fig. 5d). To clarify the association between PPP2R2B expression and tumor immune response, we performed GSVA. As shown in Fig. 7a, b, a significant difference was observed in each dataset, including antigen processing and presentation, T cell activation, alpha–beta T cell activation, and positive regulation of T-helper 1 cell cytokine production.

### PPP2R2B was associated with immune checkpoint members and antigen presentation family genes

**Fig. 6:**
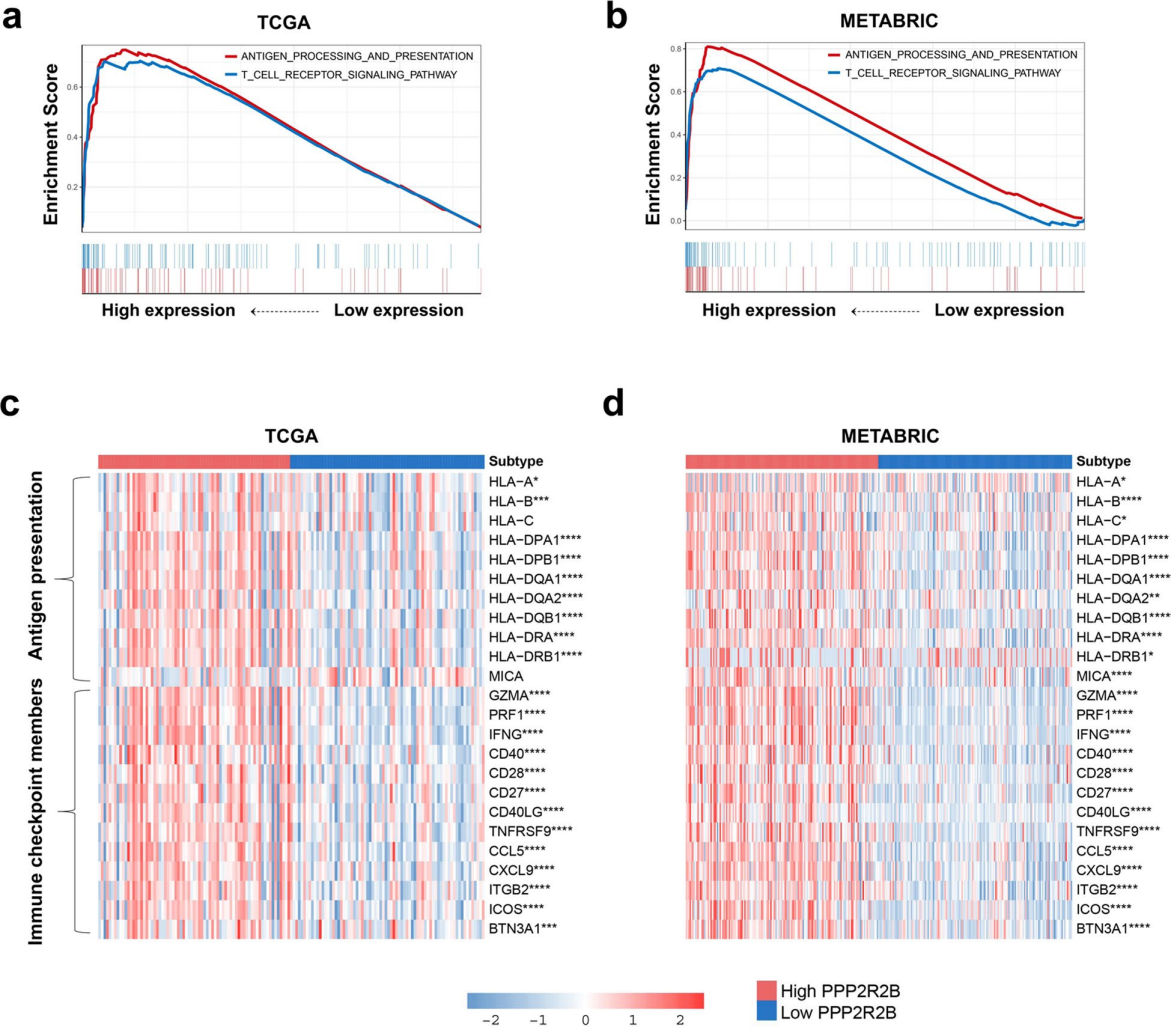
PPP2R2B was associated with immune checkpoint members and antigen presentation family genes. **a**, **b** Gene set enrichment analysis in TCGA and METABRIC, respectively. **c**, **d** The difference levels of the genes associated with antigens presentation and immune checkpoint members in TNBC and METABRIC patients with different PPP2R2B expression, respectively. *ns* Not significant, *p < 0.05, **p < 0.01, ***p < 0.001 and ****p < 0.0001

**Fig. 7:**
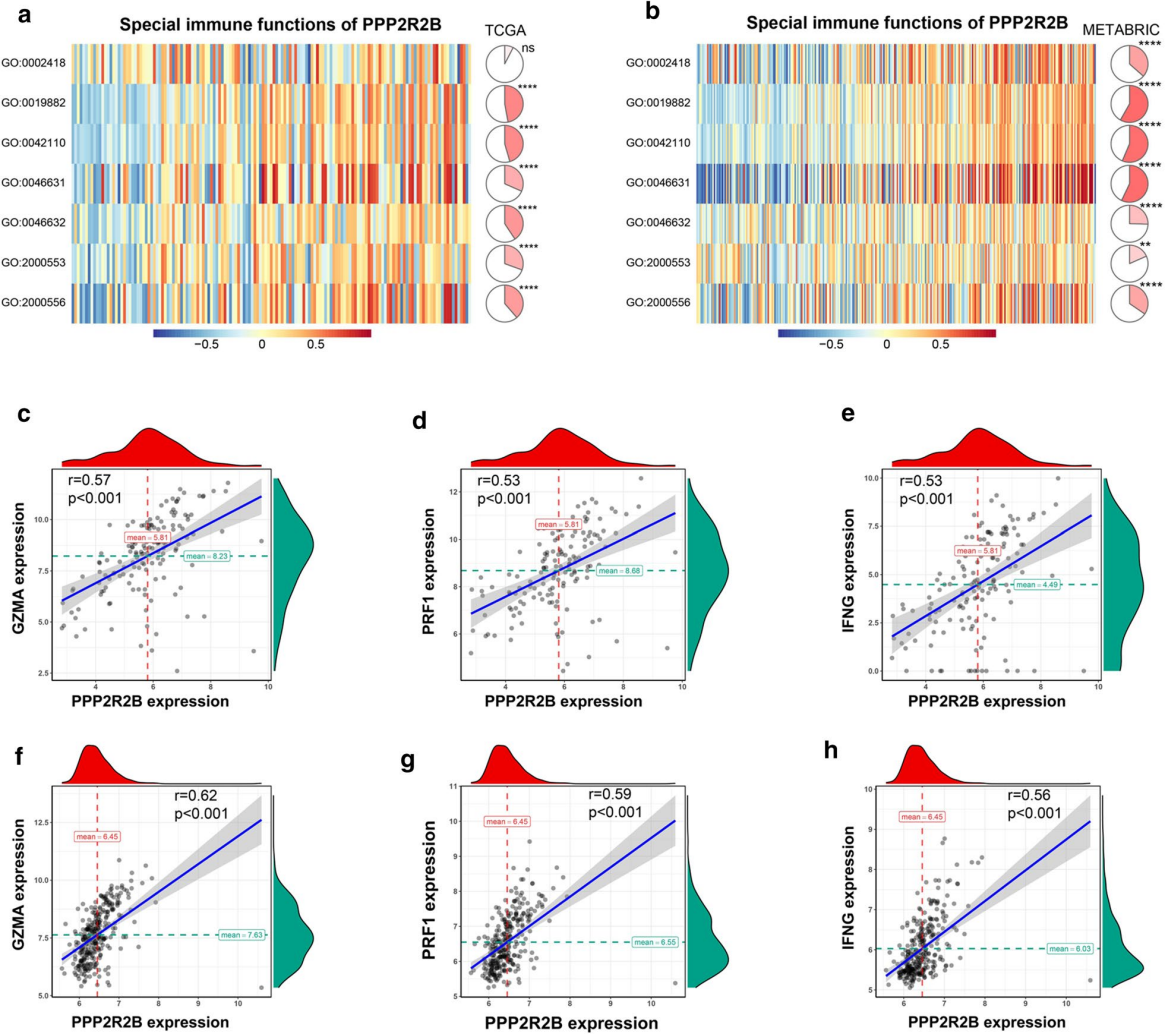
Further evaluation of PPP2R2B related immunity functions in TNBC. **a**, **b** The relationship between PPP2R2B expression and T cell immunity in TCGA and METABRIC, respectively. **c**–**e** The most related immune checkpoint family genes with PPP2R2B expression in TCGA. **f**–**h** The most related immune checkpoint family genes with PPP2R2B expression in METABRIC. GO:0002842 positive regulation of T cell mediated immune response to tumor cell; GO:0019882 antigen processing and presentation; GO:0042110 T cell activation; GO:0046631 alpha–beta T cell activation; GO:0046632 alpha–beta T cell differentiation; GO:2000553 positive regulation of T-helper 2 cell cytokine production; GO:2000556 positive regulation of T-helper 1 cell cytokine production. ns (Not significant), *p < 0.05, **p < 0.01, ***p < 0.001 and ****p < 0.0001

Here, GSEA was conducted to determine that the genes in antigen processing and presentation pathway and T cell receptor signaling pathway were enriched in the high expression group of PPP2R2B (Fig. 6a, b). Subsequently, we further investigated the relationship of genes related with immune checkpoints and antigen presentation in two subgroups. As presented in Fig. 6c, d, immune checkpoint inhibitor genes were highly enriched in the high expression group. Moreover, most genes (except for HLA-C and MICA) related with antigen presentation were strikingly activated in the high expression group. Of note, we focused on three strongly positively related genes in TCGA and METABRIC datasets, including GZMA, IFNG, and PRF1, which played a crucial role in anti-tumor immune of T lymphocytes (Fig. 7c–h). These findings suggested that PPP2R2B upregulation may associate with improved anti-tumor immune response of T-lymphocyte, whereas PPP2R2B downregulation could contribute to tumor immune evasion.

### PPP2R2B downregulation promoted M1 phenotype polarization and migration capability

**Fig. 8:**
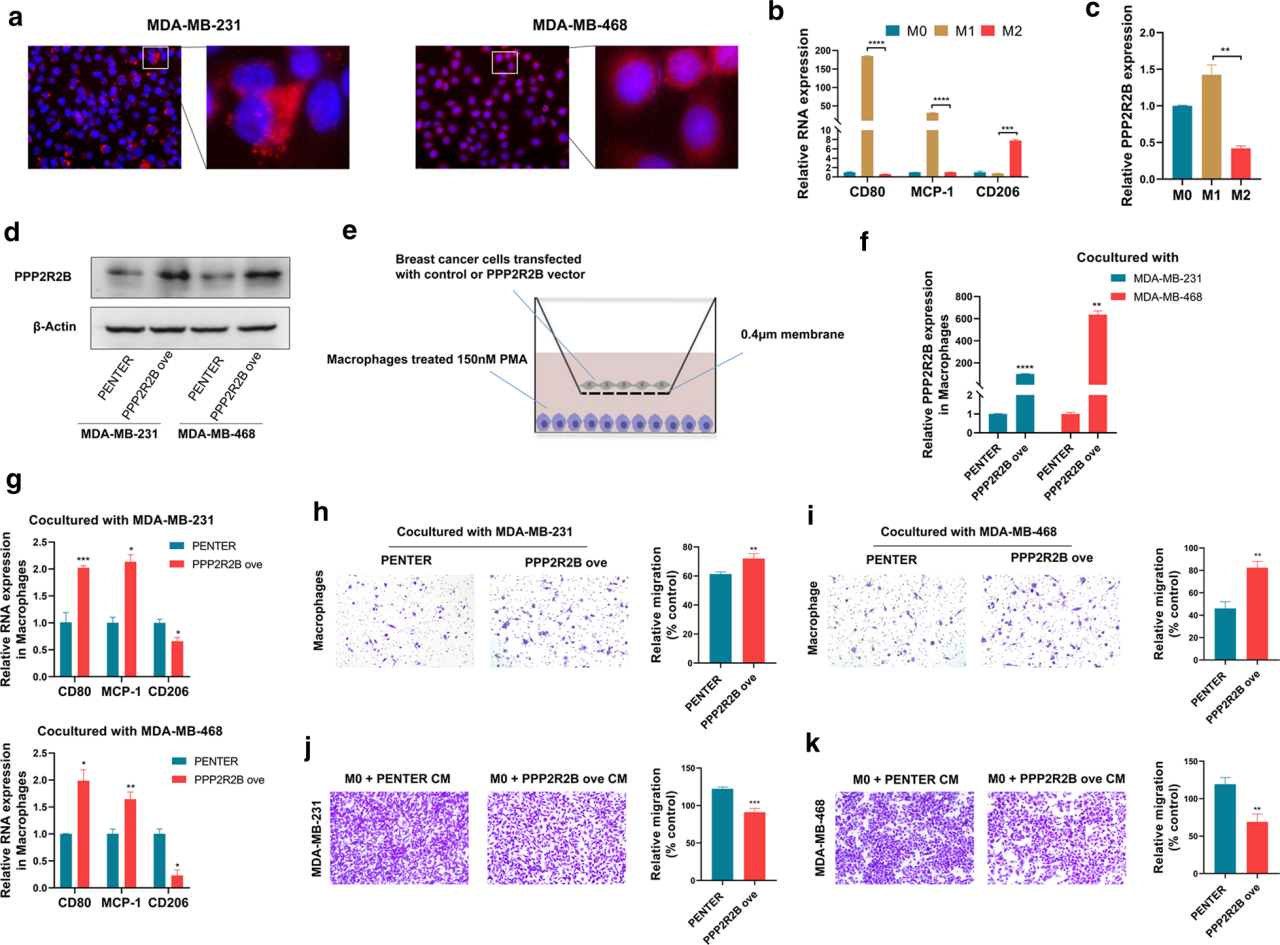
PPP2R2B downregulation promoted M1 phenotype polarization and migration capability.** a** Immunofluorescence assay. **b** Q-PCR assay validated whether the THP-1 monocytes had been successfully polarized into M1 or M2 macrophages. **c** Q-PCR assay identified that PPP2R2B was significantly upregulated in M1 macrophages compared with M2 macrophages. **d** Western blotting evaluated the efficacy of PPP2R2B expression. **e** The schematic diagram of co-culture system. **f** Cocultured macrophages exhibited different PPP2R2B expression. **g** Altered expression of polarization-specific genes in macrophages cocultured with TNBC cells transfected with empty or PPP2R2B overexpressing vector, respectively. upper panel: Cocultured with MDA-MB-231 cells; lower panel: Cocultured with MDA-MB-468 cells. **h**, **i** Cocultured macrophage was selected to perform the transwell assay. **j**, **k** Transwell assay identified that PPP2R2B-educated macrophages could significantly suppress TNBC cells migration. ns (Not significant), *p < 0.05, **p < 0.01, ***p < 0.001 and ****p < 0.0001

To further validate the effect of PPP2R2B on immune activity, we performed in vitro experiments based on macrophages. Immunofluorescence assay showed that PPP2R2B protein was predominantly located in the cytoplasm of MDA-MB-231 and MDA-MB-468 cells (Fig. 8a). THP-1 monotypes treated with PMA were differentiated to M1 or M2 macrophages by treatment with LPS/INF-γ or IL-4/IL13, respectively. As shown in Fig. 8b Q-PCR assay validated that the THP-1 monotypes were successfully polarized into M1 or M2 macrophages. Subsequently, we observed that PPP2R2B was significantly upregulated in M1 macrophages compared with M2 macrophages (Fig. 8c). Following empty or PPP2R2B overexpressing vector was transfected into MDA-MB-231 and MDA-MB-468 cells for 48 h, the efficacy of PPP2R2B expression was evaluated via western blotting in two TNBC cell lines (Fig. 8d). As depicted in Fig. 8e, co-culture assay was carried out after PMA treated TPH-1 cells for 24 h. Through 72 h of co-culture, macrophages cultured with PPP2R2B-overexpressing TNBC cells exhibited significantly increased PPP2R2B expression compared to those cultured with TNBC cells transfected with empty vector (  8f). Meanwhile, mRNA expression of M1-related genes such as CD80 and MCP-1was significantly upregulated in macrophages cultured with PPP2R2B-overexpressing group compared to the control group (Fig. 8g). In contrast, M2-specific marker CD206 expression was reduced in PPP2R2B-overexpressing group (Fig. 8g), which was previously identified as a suppressor for function of cytotoxic CD8 T cells [32]. Then cocultured macrophages were selected to perform the transwell assay (Fig. 8h, i). Compared with the control group, macrophages cocultured with PPP2R2B-overexpressing TNBC cells presented an increased migration capability, indicating that PPP2R2B could promote M1 phenotype polarization, and enhance its migration capability. To further investigate whether PPP2R2B-induced M1 macrophages exhibited anti-tumorigenic features, we firstly cultured macrophages with medium supplemented with supernatants collected from PPP2R2B-overexpressing or control TNBC cells. Conditioned medium (CM) from educated macrophages was harvested 72 h later, and then used to treat TNBC cells. Subsequent transwell assay identified that PPP2R2B-educated macrophages could significantly suppress TNBC cells migration (Fig. 8j, k). Moreover, we examined whether PPP2R2B could directly suppress TNBC malignant features in ways independent of the immune system. Our results demonstrated that PPP2R2B upregulation could remarkably reduce resistance to doxorubicin and migration capability of TNBC cells (Additional file 8: Figure S3a–d).

### Dysregulation of PPP2R2B exhibits distinct phenotype characters in genomic level

**Fig. 9:**
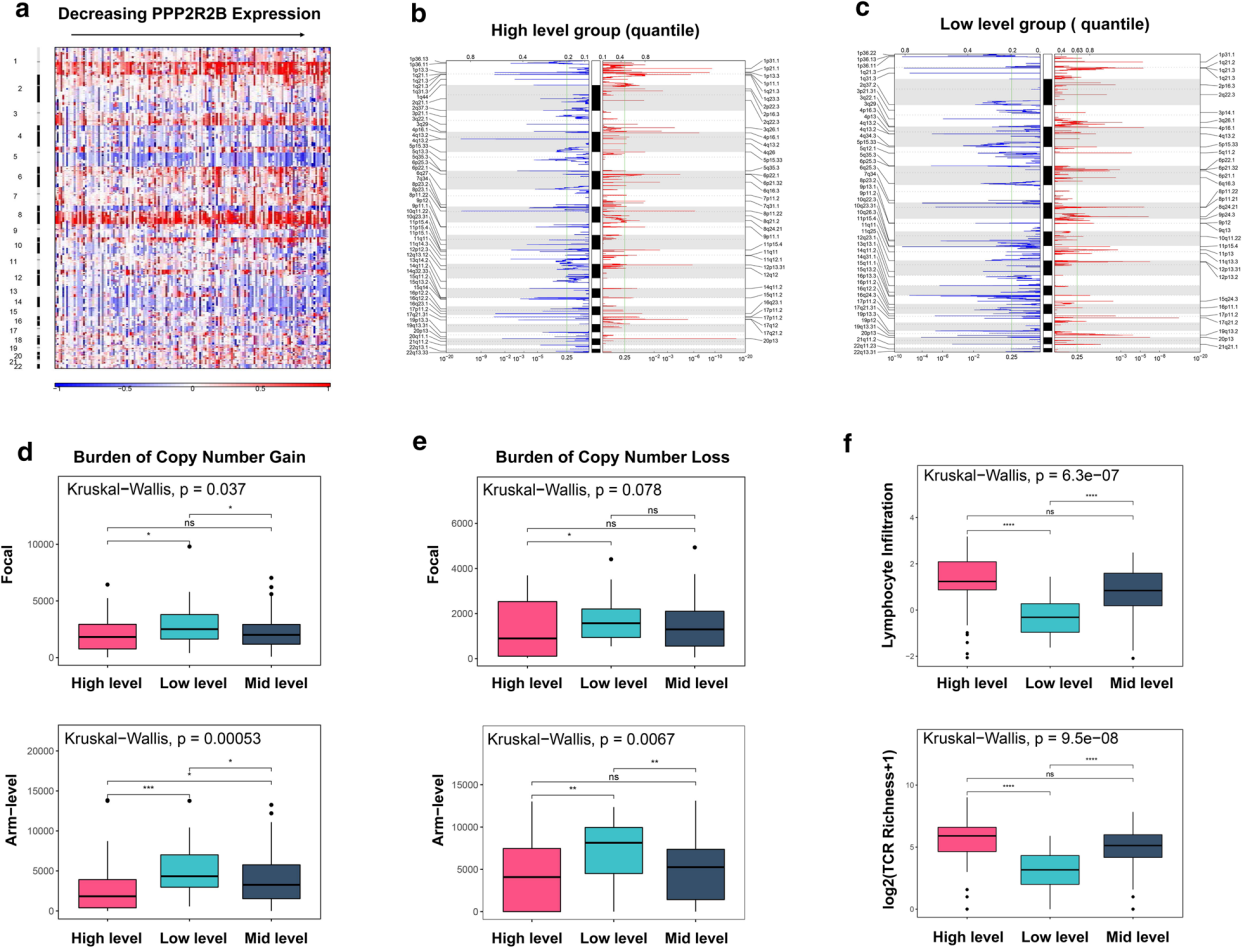
Distinct genomic profiles of copy number alterations (CNAs). **a** CNAs at arm level. **b** CNAs of high level group at focal level. **c** CNAs of low level group at focal level. **d** Burden of copy number gain at arm or focal level. **e** Burden of copy number loss at arm or focal level. **f** The relationship between PPP2R2B expression and lymphocytes infiltration or T cell receptor (TCR) richness. ns (Not significant), *p < 0.05, **p < 0.01, ***p < 0.001 and **** *p* < 0.0001

To further uncover its anti-tumor molecular mechanism in TNBC, we analyzed somatic mutations and copy number alterations (CNAs) sourced from TCGA dataset. We firstly analyzed the CNAs data at the arm level. A frequent chromosome 5 deletion event (5q deletions) was observed in Fig. 9a. According to previous studies, 5q deletion event is specific trans module of basal-like subtype, which transcriptional alterations were mainly involved in cell cycle, DNA damage repair, and apoptosis [33]. Intriguingly, 5q deletion signal was strongly enriched in low PPP2R2B expression group, and increasing along with decreasing of PPP2R2B expression (Fig. 9a). This finding could well explain why PPP2R2B downregulation was more likely to associate with phenotype characters of basal-like subtype. Moreover, a trend towards a higher burden of 1q and 8q gain was observed in low PPP2R2B expression compared to high PPP2R2B expression (Fig. 9a). According to previous reports, 1q and 8q gain was frequently associated with tumor initiation and progression in several cancer types, containing breast cancer [34, 35].

To assess CNAs at focal genomic level, we stratified TNBC patients into three subgroups according to quartile of PPP2R2B expression. In high-level group, CD247 (1q23.3) represented significant amplification peaks accompanied by PD-1 (2q37.3) and LAG3 (12p13.31) deletion peaks (Fig. 9b; Additional file 9: Table S6). In low-level group, PD-L1(9p24.1) and CD274 (9p24.1) amplification peaks were observed (Fig. 9c; Additional file 9: Table S6). Of note, copy number aberrations of these genes could contribute to tumor immune escapes. Additionally, driver oncogenes such as MYC (8q24.21), CCND1(11q13.3), and JAK2(9p24.3) in low-level group exhibited more significant amplification peak than high-level group (Fig. 9b, c; Additional file 9: Table S6). By contrast, amplification peaks of tumor suppressor genes (NF1,17p11.2; MAP2K4, 17p11.2) were detected in high-level group (Fig. 9b; Additional file 9: Table S6).

Previous study reported that the burden of copy number loss was associated with poor response to anti-CTLA-4 blockade [36]. We then explored the relationship between copy number gain or loss status and PPP2R2B expression (Fig. 9d, e). Our results showed that the high-level group have a lower CNAs burden (gain or loss) compared with the low-level group. Meanwhile, low CNAs burden (high PPP2R2B expression) was significantly related with increased lymphocytes infiltration and T-cell receptor (TCR) richness (Fig. 9f). TCR was previously reported to enhance the abundance of T cells and initiate anti-tumor immune response via recognizing cancerous cells [37]. In general, increased richness of TCR in tumor region is associated with an improved immunotherapeutic response [38]. Taken together, PPP2R2B can act as a promising biomarker for predicting which patients will response to immune checkpoint blockade, and further guide personalized immunotherapeutic strategy for TNBC patients.

**Fig. 10:**
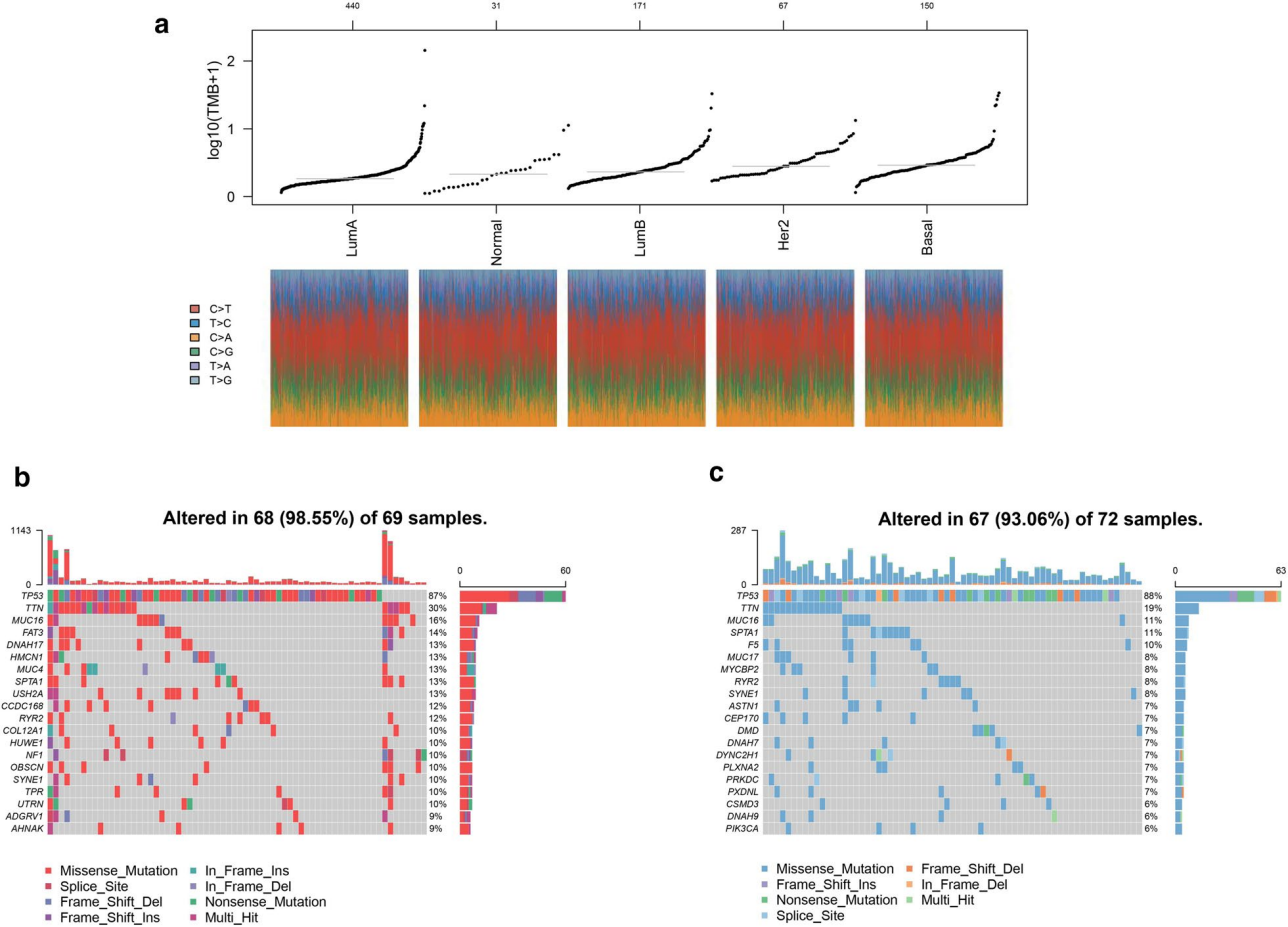
Distinct genomic profiles of somatic mutation. **a** TMB difference among distinct breast cancer subtypes. **b** Mutation profiles in high PPP2R2B expression group. **c** Mutation profiles in low PPP2R2B expression group

Given that tumor-specific antigens generated by somatic mutation could drive T cell infiltration and influence immunotherapeutic clinical outcomes [39, 40], we investigated TNBC somatic mutations sourced from TCGA. Figure 10a revealed that basal-like subtype had higher tumor mutation burden (TMB), which could initiate tumorigenesis and, conversely, activate CD8 T cells to improve anti-tumor responses [41–43] .In this analysis, TNBC patients were classified into two subgroups based on PPP2R2B median expression. Subsequent analysis indicated that high expression group appeared higher mutation frequency than low expression group (98.55 vs 93.06%) (Fig. 10b, c). Moreover, we focused on a significant difference associated with TTN mutation between two subgroups, 30% in high group and 19% in low group (Fig. 10b, c).

### PPP2R2B downregulation was associated with promoter hypermethylation

**Fig. 11:**
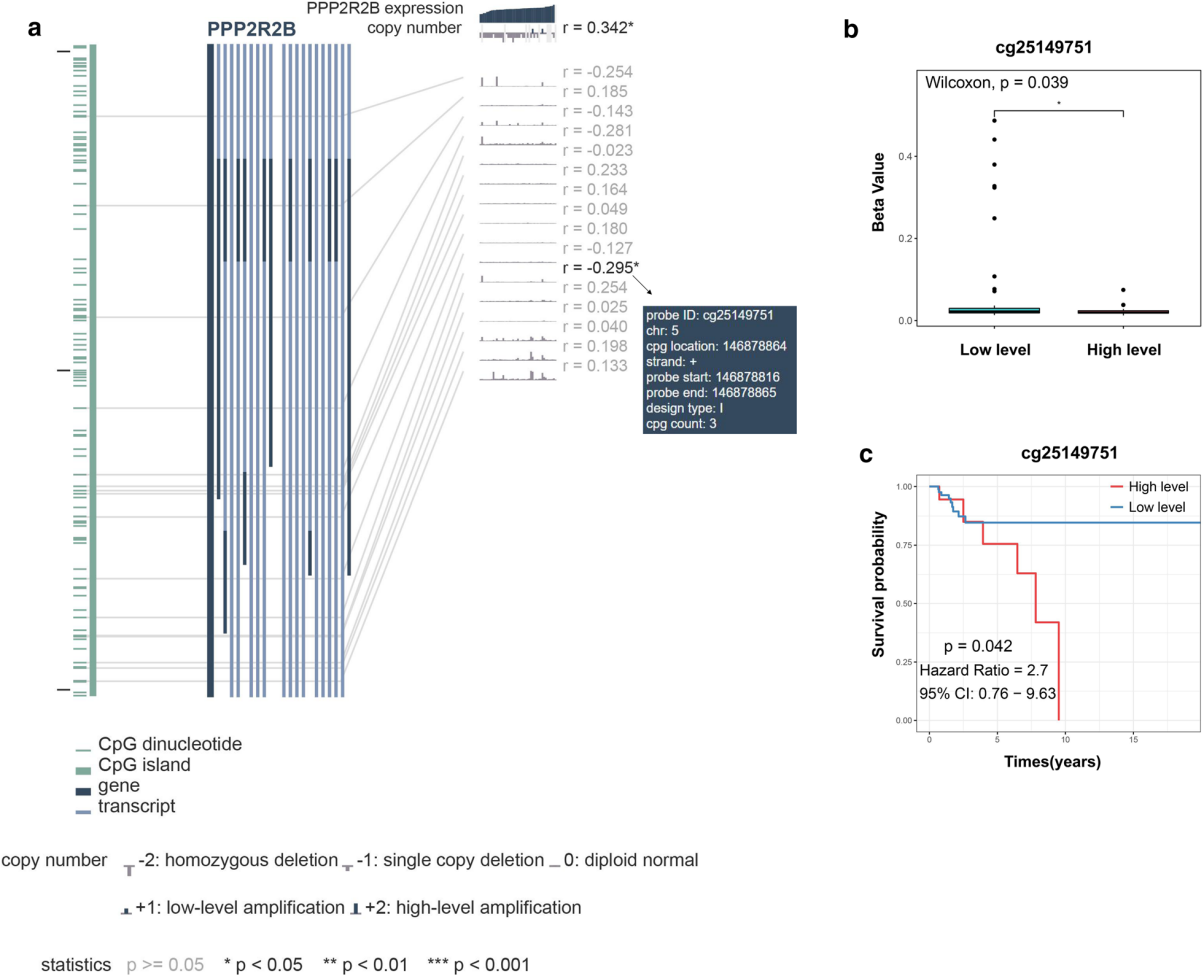
PPP2R2B downregulation was associated with promoter hypermethylation. **a** DNA methylation level in PPP2R2B promoter region. **b** Methylation difference between high- and low- subgroups. **c** Kaplan‐Meier survival analysis associated with methylation level

To explore the mechanism of PPP2R2B downregulation in TNBC, we assess the relationship between PPP2R2B expression and methylation of PPP2R2B promoter region. As shown in Fig. 11a, methylation level of CpG island (cg25149751) located in PPP2R2B promoter had significantly negative correlation with PPP2R2B expression. Wilcoxon test further demonstrated that difference between high- and low-level subgroups of PPP2R2B expression was statistically significant (Fig. 11b). Moreover, high level of cg25149751 methylation predicted shorter overall survival in TNBC patients (Fig. 11c). These findings suggested that promoter region methylation may silence PPP2R2B promoter to downregulate PPP2R2B expression.

## Discussion

Immune evasion is regarded as an important hallmark of cancer, and play a crucial role in tumor initiation and progression [44, 45]. Previous studies revealed that immune evasion is mainly resulted from immunosuppression microenvironment and tumor immunoediting, which make tumor cells to escape from immune destruction of CD8 cytotoxic T lymphocytes (CTLs), CD4 Th1 helper T cells or natural killer (NK) cells [45, 46]. With the depth understanding of cancer immune evasion, immunotherapy have represented a promising therapeutic avenue for patients with cancer [7, 47]. Due to higher levels of PD-L1 expression, more numbers of TILs, and greater rates of nonsynonymous mutations, TNBC patients show greater immunotherapeutic potential than other molecular subtypes [8]. To date, various immunotherapeutic agents have been developed and improved in TNBC patients. Of which, immune checkpoint inhibitors represent particularly attractive, but anti-PD-1 or anti-PD-L1 antibody alone remains low response rate among TNBC patients [9]. This was partially attributed to immune suppression of anti-tumor T lymphocytes [9]. Additionally, previous reports indicated that elevated TILs levels are frequently associated with a lower risk of relapse and improved survival time in TNBC patients [3, 6]. Therefore, it is essential to further investigate molecular biomarkers associated with anti-tumor T lymphocytes, which could help develop novel therapeutic targets, predict immunotherapy response, and guide personalized strategies in TNBC treatment.

Of infiltrating immune cells, CD8 T cells function as a central player in mediating tumor specific immune response [12, 13]. In this study, we applied integrated bioinformatics analysis to develop CD8 T related signature score in TNBC. Finally, PPP2R2B was identified as a hub tumor suppressor that was frequently downregulated in TNBC tissues compared with normal breast tissues. Kaplan–Meier survival analysis revealed that the increased expression of PPP2R2B was related with TNBC patients’ improved survival time. Moreover, our results indicated that PPP2R2B expression was significantly positively correlated with the infiltration of CD8 T cells, CD4 Th1 cells, and M1 macrophages, and negatively associated with the infiltration of M0 and M2 macrophages. Q-PCR assays showed that PPP2R2B could associate with the early-onset of breast cancer, and the malignant progression of TNBC. Thus, dysregulation of PPP2R2B expression could serve as a pathologic driver of breast cancer, implicating its potential value as a candidate predictive biomarker and therapeutic target.

Phosphatases are previously reported to participate in several biology process, including immune response [16]. More specifically, phosphatases could directly or indirectly correlate with anti-tumor T cell response. In our study, the gene ontology analysis of biological functions was performed to identify that PPP2R2B could play a crucial role in tumor immune response. Subsequent GSEA and GSVA confirmed that PPP2R2B had strongly positive correlation with antigen processing and presentation and T cells activation. Since immunotherapy associated with immune checkpoints have provided clinical benefits for several patients with cancer [48, 49], we analyzed the relationship between PPP2R2B expression and immune checkpoints family members. Notably, our results suggested that PPP2R2B expression exhibited strongly positive correlation with GZMA, PRF1, and IFNG compared to other members. GZMA, PRF1, and IFNG were previously determined as immune checkpoint inhibitor genes [31]. Thereby, downregulation of their expression could restrict immune surveillance and facilitate tumor malignant progression. Besides, we observed most genes associated with antigen presentation were activated in high expression group of PPP2R2B.These findings suggested that PPP2R2B downregulation was also likely to contribute to tumor immune evasion through suppressing antigen presentation pathway.

Interestingly, PPP2R2B represented significantly positive correlation with favorable prognostic M1 macrophages, and negative correlation with unfavorable prognostic M2 macrophages. In solid tumors, tumor-associated macrophages(TAM) is one of the most abundant immune cell types, of which M2 TAM (M2 macrophages) serve as a suppressor of CTLs function [50, 51]. M2 phenotype could directly suppress full activation and recruitment of CD8 T cells, and restrain response to anti-PD-1 treatment. Conversely, M1 phenotype displays antitumor characters [52, 53]. Therefore, we conjectured that PPP2R2B may be a key regulator of macrophage polarization. Afterwards, we further validate the effect of PPP2R2B on immune activity via in vitro experiments based on macrophages. Our results showed that PPP2R2B could drive macrophage polarization towards M1 phenotype, and promote the migration capability of M1 phenotype macrophages. By contrast, M1 phenotype macrophages derived by PPP2R2B could significantly impair TNBC cells migration. Consequently, we consider that PPP2R2B downregulation may help TNBC cells to evade immune surveillance partly via regulating macrophages polarization towards an M2 phenotype.

Genomic alterations and heterogeneity have been demonstrated to contribute to resistance to immune checkpoint blockade [36, 54]. Through the further analysis of copy number alterations at genomic level, we observed that three heterogeneous alterations (5q deletion event,1q and 8q amplification events) were increasing along with decreasing of PPP2R2B expression. In particular, the increasing of 5q deletion event made patients with low level of PPP2R2B expression more inclined to heterogeneous clinical features of TNBC. In addition, the amplification peaks of oncogenes such as MYC, JAK2, and CCND1 were more significant in patients with low level of PPP2R2B expression. Meanwhile, the amplification peaks of cancer suppressors containing NF1 and MAP2K4 were detected in patients with high level of PPP2R2B expression. Besides, aberrant amplification or deletion event associated with immune checkpoint stimulator genes (CD247, CD274, PD-1, PD-L1, and LAG3) was observed in distinct subgroups, upregulation of which expression could restrict anti-tumor response and facilitate immune escapes.

According to previous reports, neoantigens derived from somatic mutation were considered as important immunotherapeutic antigens [55], and higher mutation burden could produce more clinical benefits for patients treated with immune checkpoints blockade [56].Somatic mutation analysis was performed to demonstrate that PPP2R2B upregulation was associated with higher mutation frequency in TNBC patients. More importantly, in the group with high level of PPP2R2B expression, we observed greater frequent TTN mutation. Notably, mutated TTN was previously reported as an indicator associated with a good response to immune checkpoint blockade and longer survival time in patients with solid tumors [57].

Emerging evidence has shown that methylation within the gene promoter region could contribute to tumorigenesis by silencing tumor suppressor genes [58]. Hence, we further investigate whether PPP2R2B downregulation in TNBC was associated with promoter region methylation. Consistent with previous studies [19, 20, 59], CpG island methylation located in PPP2R2B promoter was also detected in TNBC samples. Meanwhile, the level of methylation was negatively related with PPP2R2B expression, and high methylation level was associated with poor survival time in TNBC patients.

This study provided a basis for the understanding of the complex interaction between PPP2R2B and tumor immunity. However, some statements such as promoter region methylation were mainly based on publicly available datasets. Given that the public sample size is limited, further experimental evidence and prospective studies with more sufficient sample size are warranted.

## Conclusion

In this study, we identified PPP2R2B as a robust tumor suppressor in TNBC by systemic bioinformatics analyses. Evidences sourced from the genome, transcriptome, and in vitro experiments supported that PPP2R2B downregulation could help TNBC cells to evade immune surveillance via suppressing anti-tumor immune response. Overall, PPP2R2B could act as a promising biomarker for TNBC, and help predict immunotherapeutic response and guide personalized strategies in TNBC treatment.

## Supplementary Information

**Additional file 1: Table S1.** The inclusion patient ids.

**Additional file 2: Table S2.** Overlapping genes inTCGA and METABRIC.

**Additional file 3: TableS3.** Primary filter via univariate Cox regression analysis based on TCGA dataset.

**Additional file 4: Table S4.** Primers used for Q-PCR.

**Additional file 5: Figure S1. a, b** Kaplan‐Meier survival analysis for six immune signature genes.

**Additional file 6: Figure S2.** **a** Clustering dendrogram of mRNAs. **b**, **c** The highly related genes with immune signature score in TCGA and METABRIC, respectively. **d** PPP2R2B appeared more frequently than other genes across iterations analysis.

**Additional file 7: TableS5.** Spearman correlation in TCGA and METABRIC (R>0.3).

**Additional file 8: Figure S3.** **a**, **b** IC50 value for doxorubicin in TNBC cells transfected by empty or PPP2R2B overexpressing vector. **c**, **d** Transwell assay showed that PPP2R2B remarkably inhibited TNBC cells migration.

**Additional file 9: Table S6.** The alteration peaks of CNAs in breast cancer with different PPP2R2B expression.
