# Supplementary material for: PPP2R2B downregulation is associated with immune evasion and predicts poor clinical outcomes in triple-negative breast cancer
Source: Cancer Cell Int. 2021 Jan 6;21:13. doi: 10.1186/s12935-020-01707-9 (PMC7788839; doi:10.1186/s12935-020-01707-9)
Supplement: Supplementary file 4 — Additional file 4: Table S4. Primers used for Q-PCR. [file 12935_2020_1707_MOESM4_ESM.docx]

**Table S4. Primers used for Q-PCR**

| **Gene** | **Forward （5’-3’）** | **Reverse （5’-3’）** |
| --- | --- | --- |
| PPP2R2B | ATGACTACCTCCGCAGCAAGCT | CATCACGCTTGGTGTTTCTGTCG |
| CD80 | CTCTTGGTGCTGGCTGGTC | GCCAGTAGATGCGAGTTTG |
| MCP-1 | AGAATCACCAGCAGCAAGTG | CCTGAACCCACTTCTGCTTG |
| CD206 | AGCCAACACCAGCTCCTCAAGA | CAAAACGCTCGCGCATTGTCCA |
| Actin | CATGTACGTTGCTATCCAGGC | CTCCTTAATGTCACGCACGAT |
