# Supplementary figures and images for: PPP2R2B downregulation is associated with immune evasion and predicts poor clinical outcomes in triple-negative breast cancer
Source: Cancer Cell Int. 2021 Jan 6;21:13. doi: 10.1186/s12935-020-01707-9 (PMC7788839; doi:10.1186/s12935-020-01707-9)

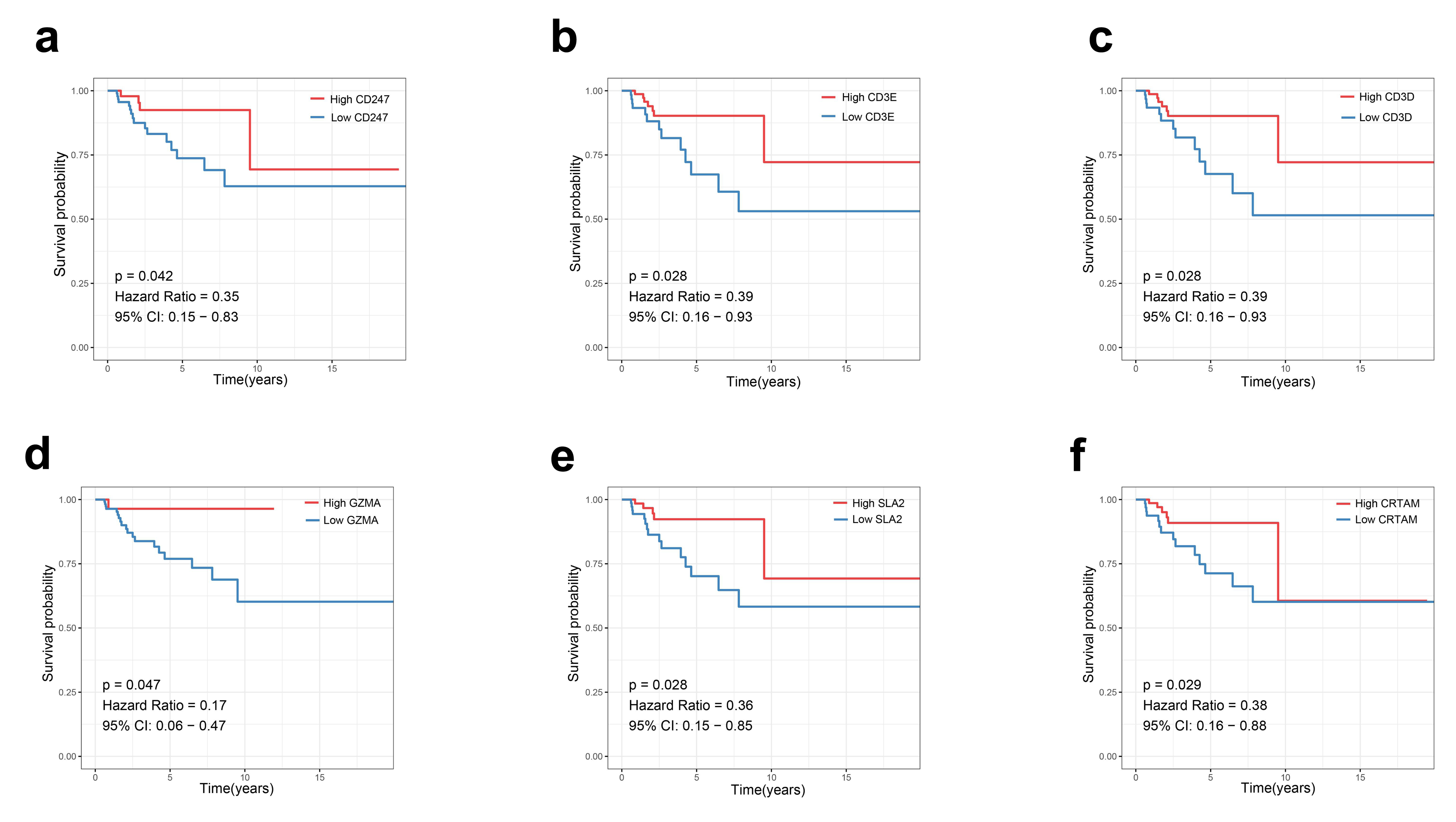

Supplement: Supplementary file 5 — Additional file 5: Figure S1. a, b Kaplan‐Meier survival analysis for six immune signature genes. [file 12935_2020_1707_MOESM5_ESM.tif]

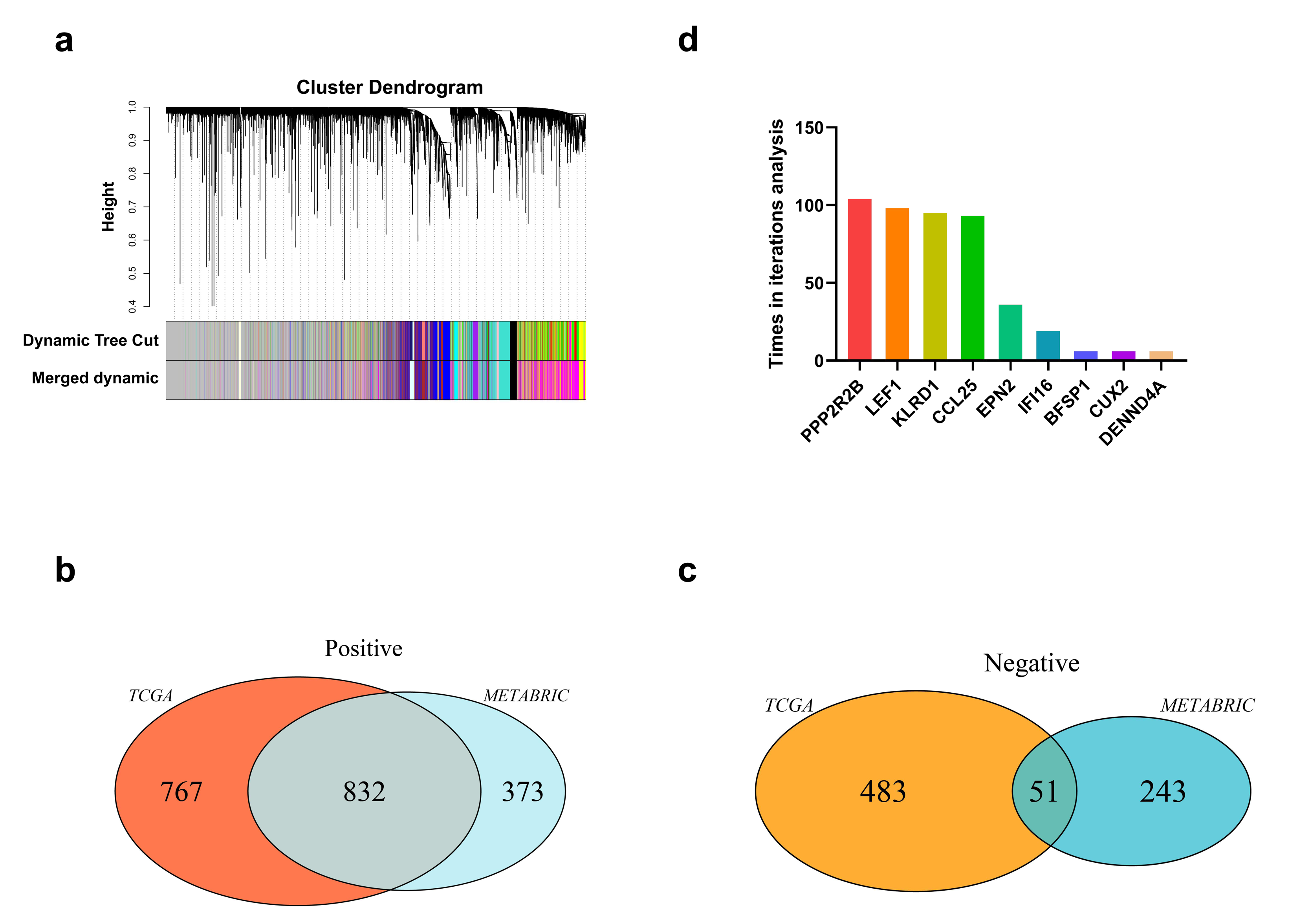

Supplement: Supplementary file 6 — Additional file 6: Figure S2. a Clustering dendrogram of mRNAs. b, c The highly related genes with immune signature score in TCGA and METABRIC, respectively. d PPP2R2B appeared more frequently than other genes across iterations analysis. [file 12935_2020_1707_MOESM6_ESM.tif]

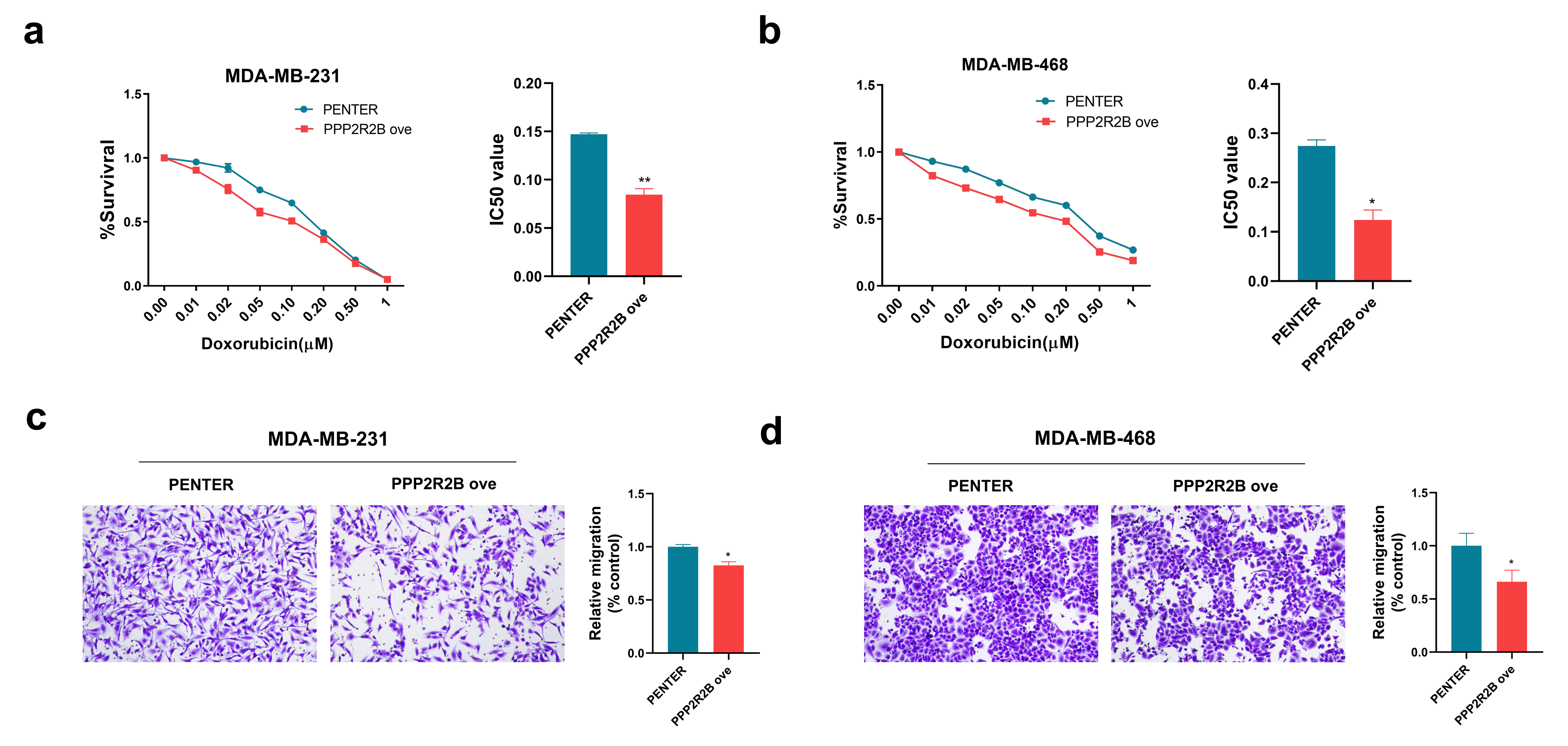

Supplement: Supplementary file 8 — Additional file 8: Figure S3. a, b IC50 value for doxorubicin in TNBC cells transfected by empty or PPP2R2B overexpressing vector. c, d Transwell assay showed that PPP2R2B remarkably inhibited TNBC cells migration. [file 12935_2020_1707_MOESM8_ESM.tif]
